# Anti-proliferative and Anti-metastatic Potential of Curcumin Analogue, Pentagamavunon-1 (PGV-1), Toward Highly Metastatic Breast Cancer Cells in Correlation with ROS Generation

**DOI:** 10.15171/apb.2019.053

**Published:** 2019-08-01

**Authors:** Edy Meiyanto, Herwandhani Putri, Yonika Arum Larasati, Rohmad Yudi Utomo, Riris Istighfari Jenie, Muthi Ikawati, Beni Lestari, Noriko Yoneda-Kato, Ikuko Nakamae, Masashi Kawaichi, Jun-Ya Kato

**Affiliations:** ^1^Department of Pharmaceutical Chemistry, Faculty of Pharmacy, Universitas Gadjah Mada, Yogyakarta 55281 Indonesia.; ^2^Cancer Chemoprevention Research Center, Faculty of Pharmacy, Universitas Gadjah Mada, Yogyakarta 55281 Indonesia.; ^3^Laboratory of Tumor Cell Biology, Nara Institute of Science and Technology, Japan.; ^4^Laboratory of Gene Function in Animals, Nara Institute of Science and Technology, Japan.

**Keywords:** Anti-proliferative, Metastatic inhibitor, PGV-1 (curcumin analogoe), Reactive oxygen species (ROS), The 4T1 cells

## Abstract

***Purpose:*** Pentagamavunon-1 (PGV-1) is a curcumin analogue that shows cytotoxic activity in various cancer cells. In this study, we evaluated the effect of PGV-1 on a highly metastatic breast cancer cell line, the 4T1 cells, as an anti-metastatic and anti-proliferative agent.

***Methods:*** Cell viability was evaluated using MTT assay; while cell cycle profile, apoptosis incidence, and ROS intracellular level were determined by flow cytometry. Cell senescence was observed under senescence-associated-β-galactosidase (SA-β-gal) staining assay. The expression of matrixmetalloproteinase-9 (MMP-9) was determined using immunoreaction based-ELISA, while other proteins expression were detected using immunoblotting.

***Results:*** Curcumin and PGV-1 showed cytotoxic effects on 4T1 cells with IC50 value of 50 and 4 µM, respectively. The cytotoxic activity of PGV-1 was correlated to the induction of G2/M cell cycle arrest and cell senescence. Furthermore, PGV-1 increased the accumulation of intracellular ROS level. We also revealed that PGV-1 bound to several ROS-metabolizing enzymes, including glyoxalase I (GLO1), peroxiredoxin 1 (PRDX1), N-ribosyldihydronicotinamide: quinone reductase 2 (NQO2), aldo-keto reductase family 1 member c1 (AKR1C1). As an antimetastatic agent, PGV-1 showed less inhibitory effect on cell migration compared to curcumin. However, PGV-1 significantly decreased MMP-9 protein expression in a dose-dependent manner suggesting it still potent to inhibit metastatic cells.

***Conclusion:*** Overall, our findings suggest that PGV-1 is potential to be developed as an antiproliferative and anti-metastatic agent.

## Introduction

**Figure 1:**
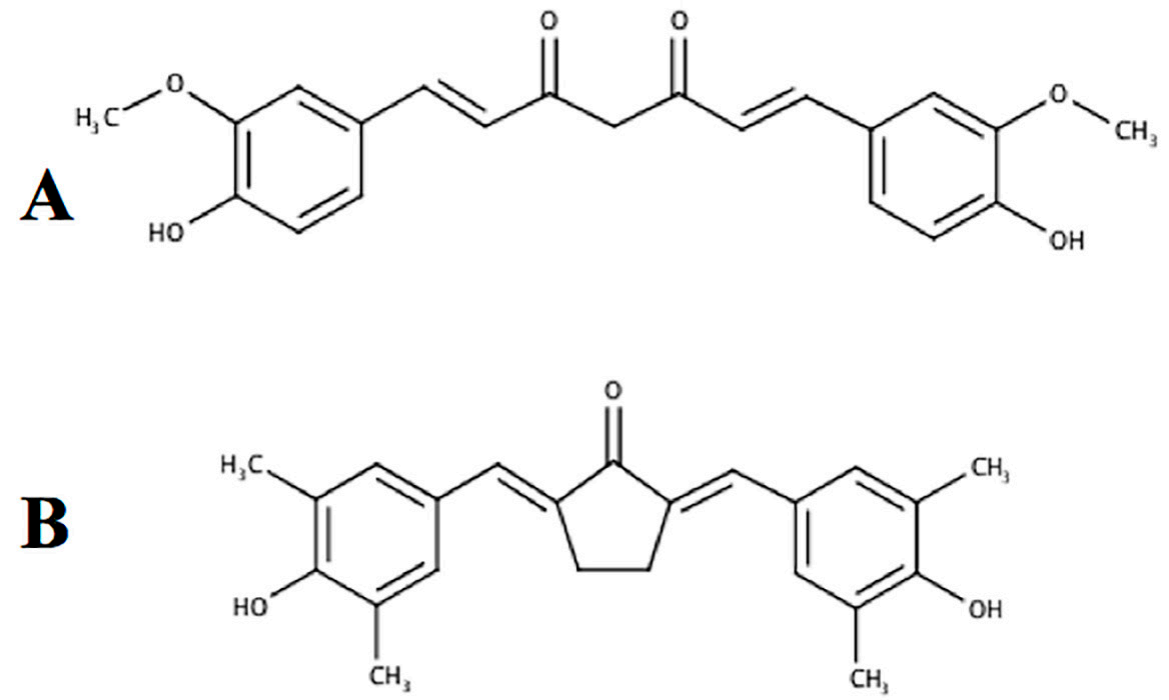
**The structure of curcumin and PGV-1.**(A) Curcumin ((1E,6E)-1-(4-hydroxy-3-methoxyphenyl)-7-(3-methoxy-4-methylphenyl)hepta-1,6-diene-3,5-dione), (B) PGV-1((2E,5E) -2- [ (4-hydroxy- 3,5-dimethylphenyl) methylidene]-5-[(3-methoxy-4,5-dimethylphenyl) methylidene]cyclopentan-1-one)

As a complex disease with the complexity of molecular based-physiological alterations, cancer challenges scientist to develop a new anti-cancer drug with specific mechanism.^1^ Previously, we reported a new curcumin analogue, namely PGV-1 (Figure 1) to have potential anti-cancer activities against several cancer cells.^2,3^ Our studies showed that curcumin and pentagamavunon-1 (PGV-1) inhibit the activity of nuclear factor kappa B (NFκB) *in vitro* and interact with the HER2 receptor *in silico*.^4^ Furthermore, we also found that PGV-1 increases the cytotoxicity of 5-fluorouracil (5-FU) in WiDR colon cancer cells.^5^ Here, we report the anti-cancer activity of PGV-1 against metastatic cancer cells, 4T1 cells, to obtain a more widely application of PGV-1 to the malignant tumors with a specific target of mechanism.

We use 4T1 cells that are characterized by the highly proliferative and metastatic activities.^6,7^ This highly metastatic cancer cell line is a suitable model to explore the new anti-cancer agent targeting on cell proliferation and metastasis. Indeed, there are numerous molecular pathways underlying the proliferative and metastatic phenomenon involving tightly regulation of energy metabolism.^8^ In this regard, intracellular reactive oxygen species (ROS) level is getting a highlight as a target of anti-cancer strategy.^9^ Interestingly, we found a new anticancer mechanism of curcumin through the ROS metabolic pathway. Curcumin exhibits cytotoxic activity in K562 cells, a leukemic cell line, by inhibiting multiple antioxidant enzymes, subsequently increasing intracellular ROS level and inducing cancer cell death.^10^

Curcumin, a well-known chemopreventive agent, is a potent tyrosine kinase inhibitor and shows inhibitory activity toward HER2^11^ ; as well as inhibits the activation of NFκB.^12^ PGV-1 (Figure 1), a curcumin analogue, is promising to be developed as an anticancer agent. PGV-1 differs from curcumin in its chemical core, which is cyclopentanone instead of di-ketone. Hence, PGV-1 is expected to have a better stability and activity compared to curcumin.^2^ PGV-1 performs better cytotoxic and anti-inflammatory activities compared to curcumin.^13,14^ In this study, we observed the anti-cancer effect of PGV-1 involving the ROS metabolic pathway and the correlation to some physiological changes. These findings will be important as the basic exploration in clinical application for metastatic breast cancers.

## Materials and Methods

### Cells culture

The 4T1 cells were kindly provided by Prof. Masashi Kawaichi, MD. PhD. Laboratory of Gene Function in Animal, Nara Institute of Science and Technology (NAIST), Japan. The cells were maintained in DMEM medium with 10% v/v FBS (Sigma), HEPES (Sigma), sodium bicarbonate (Sigma), 150 IU/mL penicillin, and 150 µg/mL streptomycin (Gibco) and 1.25 µg/mL fungizone (Gibco).

### Curcumin

Curcumin was synthesized by Curcumin Research Center (CRC) and PGV-1 was synthesized by Cancer Chemoprevention Research Center (CCRC), Faculty of Pharmacy, Universitas Gadjah Mada; and purified to gain the yield of 90 and 95 %, respectively.

### Cytotoxic assay

Cells were grown in 96-well plates (1x10^4^cells/well) and divided into untreated and treated group. A series of concentration of curcumin or PGV-1 were diluted in the culture medium. After 24h of incubation, medium was removed and cells were washed using phosphate buffer saline (PBS) (Sigma). About 5 mg/mL of 3-(4,5-dimethylthiazol-2-yl)-2,5-Diphenyltetrazolium Bromide (MTT) reagent (Sigma) in PBS was diluted in Dulbecco’s Modified Eagle Medium (DMEM) (1:9) and 100 µL of reagent was added to each well. After 3h of incubation, the reaction was stopped by the addition of sodium dodecyl sulfate (SDS) 10% in HCl 0.01 N. The plate then was incubated overnight at room temperature and dark place. To make sure that the formazan salt was dissolved, the plate was shaken for 10 minutes and the absorbance was measured using ELISA reader at λ 595 nm. The cells viability based on the absorbances and were plotted into semi log graph to obtain the linear regression then the IC_50_ values were determined using Excel MS Office 2007.

### Scratch wound healing assay

Cells (7.5x 10^4^cells/well in 500 µL of culture medium supplemented with 10% FBS) were seeded in 24 well-plate. After 24 hours, cell were starved by replacing the medium with culture medium supplemented with 0.5% FBS for 24 hours. After starvation, the cells were scratched with sterile yellow tip in a straight line and treated with 2 mL of either curcumin or PGV-1. The cells were documented at 0, 18, 24, and 42 hours after treatment by digital camera (Nikon, Japan). The results were analyzed by ImageJ software and presented as percent closure, then statistically analyzed by using SPSS 17.0.

### MMP-9 enzyme-linked immunosorbent assay

The 4T1 cells (3x10^5^ cells/well) were grown on 6 well plate and treated with PGV-1 2.5, 5, and 10 µM for 24 hours. Medium of the cells were collected as lysate protein and was used as sample for Mouse Total MMP-9 Immunoassay according to the manufacture (R&D system). Each sample was mix with MMP-9 antibody, substrate solution, and stop solution then determined the optical density using a microplate reader set to 450 nm to obtain the relative expression level based on the absorbances. The data were statistically analyzed by one-way ANOVA by using SPSS 17.0.

### Cell cycle analysis and western blot of cell cycle protein

Cell cycle analysis was performed by using propidium iodide (PI)-staining flowcytometry. Cells (5×10^4^cells/well in 6-well plate) were seeded and treated with PGV-1 (2 and 4 μM) for 24 hours. On the next day, cells were harvested, washed with PBS, and fixed gently (drop by drop) in 70% cold ethanol. The cells were stained with the staining solution containing 1 mg/mL PI, 10 mg/mL RNase and 0.1% (v/v) Triton X-100 (Merck). Cells were incubated for 5 minutes in the dark room, transferred into a flowcytometric tube, and analyzed by BD Accuri C6 flowcytometry (BD Bioscience). The protein extract of the treated cells were subjected for western blotting with cyclin-B1 antibody (Cell Signalling #41355).

### Apoptosis assay

Apoptosis assay was performed by using Annexin V-FITC/PI staining flowcytometry. Briefly, 5×10^4^4T1 cells/well in 6-well plate were incubated with either PGV-1 (2 and 4 μM) for 24 hours. The cells then were harvested (both, the trypsinized, attached and detached cells) by centrifugation and were stained by using Annexin-V-FLUOS staining kit (Roche). The cells were incubated for 10 minutes in the dark room and subjected for flowcytometry using BD Accuri C6 flowcytometer (BD Bioscience).

### Intracellular ROS level measurement

Cells (5×10^4^) were seeded on 24 well plates with DMEM culture medium (Gibco) overnight. Cells were collected by trypsin-EDTA 0.25% (Gibco), then added with 500 μL 1X supplemented buffer (PBS+FBS 10%). Cells were stained by 25 μM 2’, 7’–dichlorofluorescin diacetate (DCFDA) (Sigma) then were incubated at 37°C CO_2_ 5% for 30 min. Cells were treated with Doxo 250 nM as positive control, PGV-1 (3 and 6 μM), and Cur (25 and 50 μM), then were incubated at 37°C CO_2_ 5% for 4 h. ROS level was measured by BD Accuri C6 flowcytometer (BD Bioscience).

### Senescence-associated β-galactosidase assay

Cells (5×10^4^) were grown in 6-well plate and treated with PGV-1 (3 and 6 μM). After 24 h, cells were washed with PBS and fixed by using 2% formaldehyde - 0.2% glutaraldehyde for 10 minutes. Cells were then washed again using PBS 1X then stained by using X-gal solution containing 0.2% 5-bromo-4-chloro-3-inolyl-β-D-galactoside (Sigma), 40 mM PBS 2X (pH 6.0), 5 mM K4Fe(CN)6, 5 mM K3Fe(CN)6 and 2 mM MgCl_2_. Cells were incubated for 3-4 days and observed under inverted microscope (100X magnification). The β-D-galactosidase positive cells were quantified using ImageJ software.

### Autophagy assay

Cells (2×10^5^) were seeded in 6-well plate and treated with PGV-1 (3 and 6 μM) for 24 h. Cells were stained with Green Detection Reagent provided by Autophagy Detection Kit (Abcam) based on manufacture procedure. Cells then were analyzed by BD Accuri C6 flowcytometry (BD Bioscience).

### Pulldown assay and western blot analysis

Approximately 120 mg of epoxy-activated sepharose beads (E6754 Sigma) were washed in dH_2_O for 5 times. Beads were then mixed with curcumin and PGV-1 solution in a coupling buffer (20 mM curcumin or PGV-1 in 50% dimethylformamide/0.1 M Na_2_CO_3_/10 mM NaOH) as described in Larasati et al.^10^ The lysates from HEK293T cells expressing HA-tagged were prepared in EBC lysis buffer (50 mM Tris-HCl pH 8.0, 500 mM NaCl, 0.5% NP40) supplemented with 0.1 mM NaF, 0.1 mM Na_3_VO_4_, 10 mM β-glycerophosphate, 1 mM PMSF, and 2 KIU aprotinin. About 10 μL of PGV-1 beads and curcumin beads were mixed with 10 μL of protein for 2 hours at 4°C by continuously tapping. After washing the beads with EBC buffer for 3 times, bound proteins were eluted from beads by incubation with 20 μl of 1.3 mM curcumin or PGV-1 solution for 1 hour at 4°C. The supernatant was collected, mixed with SDS-PAGE sample buffer, and boiled at 98°C for 5 minutes. Proteins bound to curcumin or PGV-1 beads were then analyzed by western blot using 12CA5/Roche antibody (anti-HA).

### Statistical analysis

All data are expressed as means ± SD or SE. Statistical analysis of the experimental data was conducted by using the one-way ANOVA analysis of variance; following Tukey post hoc using SPSS (SPSS Inc. Chicago, USA version 20). *P* values less than 0.05 were considered to be significant

## Results and Discussion

### The anti-proliferative activity of curcumin and PGV-1 in 4T1 cells

**Figure 2:**
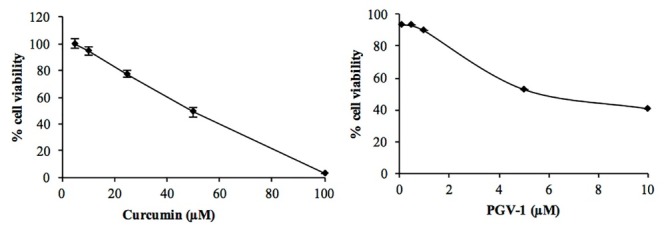
** Cytotoxic effects of curcumin and PGV-1 in 4T1 cells.** The 4T1 cells (1x10^4^cells/well) were seeded in 96 well-plate and treated with either curcumin or PGV-1 for 24 h. Cell viability was determined by using MTT assay as described in methods. The cytotoxicity of curcumin and PGV-1 was expressed by percent cell viability (mean + SD of 3 experiments). The IC_50_ values were obtained from the calculation of linear regression of concentration vs % cell viability.

The aim of this study to explore the anti-cancer activity of PGV-1, a curcumin analogue, against a highly metastasis 4T1 cells. Anti-cancer properties of curcumin and its analogues have been reviewed in several papers.^15-18^ Regardless of the potent anti-cancer activity of curcumin, one of curcumin analogue PGV-1, has not been much scrutinized yet. To date, there is no study regarding the anti-proliferative and anti-metastatic activities of PGV-1 to the highly metastatic breast cancer cells. First, we confirmed the anti-proliferative activity of PGV-1 by using MTT assay. PGV-1 exhibited a stronger anti-proliferative activity rather than curcumin with the IC_50_ value of 4 µM, while curcumin is 50 µM (Table 1 and Figure 2). In this study, we showed that PGV-1 performed a much stronger anti-proliferative activity than curcumin, indicating that PGV-1 is promising to be developed as an anti-cancer agent for metastatic cancers.

**Table 1:** IC_50_‏ values of curcumin and PGV-1 4T1 cells

**Compound**	**IC**_50_ ‏(µM‏)
Curcumin	‏50
PGV‏-1	‏4

### PGV-1 inhibits cell migration on 4T1 cells

**Figure 3:**
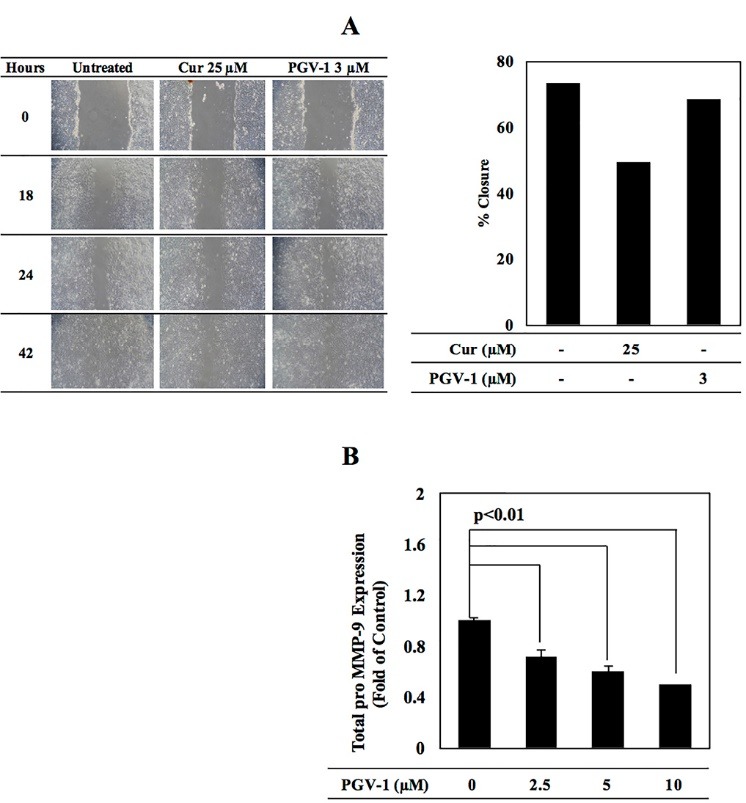
**Effect of PGV-1 cells migration of 4T1 cells**. (A) 5x10^4^4T1 cells were scratched then treated by either curcumin or PGV-1. The anti-migratory effect was quantified as % closure after 24h treatment. (B) 4T1 cells (8.5 x 10^4^cells/well) were treated with PGV-1 (2.5; 5; and 10 µM) for 24 h. Medium was collected then was analyzed by ELISA as described in methods. Data was obtained in triplicate (n=3).

In addition to evaluate PGV-1 anti-proliferative activity, we also explored the potency of PGV-1 as an anti-metastatic agent by using a highly metastatic breast cancer cells in our study. The anti-metastatic activities of curcumin and PGV-1 were screened by scratch wound healing assay and MMP-9 activity assay. The effect of PGV-1 on cell metastasis could be evaluated by determining its inhibitory activity on the migration process. Scratch wound healing assay on 4T1 cells was carried out to screen the anti-migratory effect of PGV-1. After 24 hours incubation, PGV-1 performed a slight inhibition on the migration process (Figure 3A). In addition, we found that curcumin exhibited a stronger anti-migratory activity.

We also observed the effect of PGV-1 on matrix metalloproteinase-9 (MMP-9), a protein plays an eminent role in extracellular matrix (ECM) degradation. An MMP-9-ELISA based assay was conducted to observe potential suppression of MMP-9 expression by the PGV-1 (Figure 3B). Our findings showed PGV-1 decreased MMP-9 expression in a dose-dependent manner, with the strongest suppression was caused by treatment of PGV-1 ^5^/_2_IC_50_ (10 µM).

Compared to PGV-1 treatment, curcumin performed a stronger anti-migratory activity, suggesting that PGV-1 is not potent as anti-migratory agent. Curcumin is reported to have inhibitory effect to actin polymerization.^19^ This activity may correlate to the inhibition of cell migration as actin polymerization is the important event in cells migration.^20^ In this regard, PGV-1 which possess inhibitory effect on the tubulin polymerization,^21^ but may perform a low effect to the actin interaction or most likely regardless to the molecular event of cells migration. Even though PGV-1 showed a weak inhibitory effect on the cells migration, PGV-1 significantly decrease the MMP-9 expression in a dose-dependent manner. This feature suggests that PGV-1 still exhibit a potency for metastatic cancers. Nevertheless, the cytostatic potential of PGV-1 should be considered more attention due to its strong anti-proliferative activity.

### The effect of PGV-1 on cell cycle

**Figure 4:**
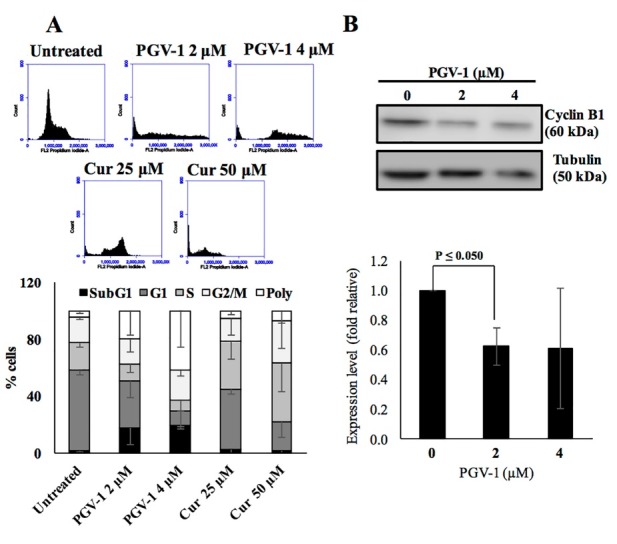
**Cell cycle modulation by PGV-1 in 4T1 Cells.** The 4T1 cells (5x10^4^cells/well) were treated by PGV-1 (2 and 4 µM) for 24h. (A) The cells then were stained by using PI and subjected to a flowcytometry and (B) the protein extracts were used for western blotting of cyclin B.

Physiological based-cytotoxicity of PGV-1 was elucidated first by cell cycle analysis. Previously, PGV-1 was reported to induce G2/M arrest on MCF-7 breast cancer and WiDr colorectal cancer cells.^4,5^ In this study, we examined the effect of PGV-1 on the cell cycle progression of 4T1 cells. After 24h of treatment, PGV-1 (2 and 4 µM) induced G2/M arrest. We also observed that PGV-1 (2 and 4 µM, 24 hours) caused sub-G1 accumulation, which possibly indicated apoptosis. Furthermore, PGV-1 treatment induced the formation of polyploid cells (26.1 %), indicating the failure of 4T1 cells dividing process at M phase (Figure 4). This phenomenon was also noted by Da’i et al,^2^ in which the treatment of PGV-1 in T47D cells leading to the formation of polyploidy cells and causing cell death. Further, we confirmed whether PGV-1 affects the main regulator of mitosis, such as cyclin B, by using western blotting. We found that PGV-1 (2 and 4 µM) decreased cyclin B expression in 4T1 cells (Figure 4B).

Treatment of PGV-1 urged cell cycle progression of 4T1 into Sub-G1 and G2/M arrest, as well as polyploid cells generation. It is consistent with our previous finding on other cell lines, that PGV-1 tend to induce G2/M arrest.^4,5^ However, further investigation is needed to understand the molecular-based mechanism of this phenomenon. Just brief insight, we found that PGV-1 decreased the cyclin B level. This finding indicates that PGV-1 acts differently compared to curcumin in inducing G2/M effect arrest. Previous study reported that curcumin tend to stabilize cyclin B through the inhibition of cdc27.^22^ The low level of cyclin B could be mediated by the active ubiquitination process or inhibition of cyclin B expression that may affect to the inhibition of Metaphase progression and will fail to undergo cytokinesis.^23^

### The effect of PGV-1 on apoptosis

**Figure 5:**
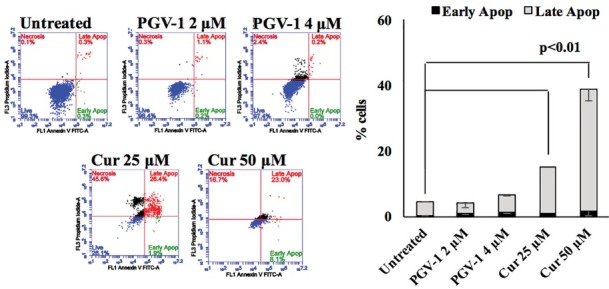
**The effect of PGV-1 on Apoptosis Induction.** The 4T1 cells (5x10^4^cells/well) were treated with PGV-1 and curcumin then were analyzed by AnnexinV-PI staining flowcytometry.

Apoptosis is closely related to cytotoxicity and cell cycle modulation. PGV-1 induces the caspase-3/7 activation and PARP cleavage, as well as induces apoptosis on T47D cells.^2,3^ PGV-1 also increased doxorubicin-induced apoptosis on MCF-7 cells.^24^ Hence, PGV-1 might induced apoptosis on 4T1 cells. However, our findings showed PGV-1 at concentration of 2 and 4 µM did not increase apoptosis compared to untreated cells (Figure 5). Therefore, PGV-1 possibly exhibited anti-proliferative activity in correlation to other mechanism.

In relation to the G2/M arrest, we note that the higher concentration of PGV-1 (4 µM) induced the accumulation of polyploid 4T1 cells. Polyploidy generation in cancer cells is the consequences of genome instability due to some disrupting agents. Polyploidy occurrence was part of a developmental program and can be acquired via different mechanisms through several steps of the cell cycle. Polyploidy also correlated with some pathological conditions as well as aging.^25^ Hence, confirmation about the apoptosis as well as senescence features under PGV-1 treatment hopefully will bring us to the clearer understanding of this event.

In this study, we showed that PGV-1 did not induce apoptosis in 4T1 cells. This phenomenon is similar with the case of curcumin that does not induce a significant apoptosis in K562 cells. In all concentration treatment, curcumin performed anti-proliferative effect and induced G2/M arrest, but apoptosis evidence was not discovered.^10^ Further, curcumin is shown to induce senescence in K562 cells.

### PGV-1 induced cells senescence

**Figure 6:**
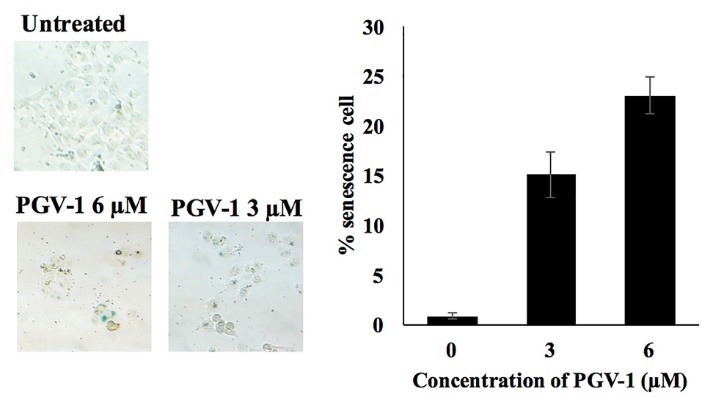
**PGV-1 induced cell senescence.** 4T1 cells were treated with PGV-1 for 3 days then were stained with β-galactosidase staining for 4 days and was monitored by light microscope.

We then determined whether PGV-1 induces cell senescence, a complex physiological process characterized by the cell neither proliferate nor die.^26^ We used senescence-associated β-galactosidase staining assay (SA-β-gal staining assay) and treated with PGV-1 at the concentration of 3 and 6 µM. We found that PGV-1 (3 and 6 µM) induced senescence in 4T1 cells (Figure 6).

### The effect of PGV-1 on autophagy

**Figure 7:**
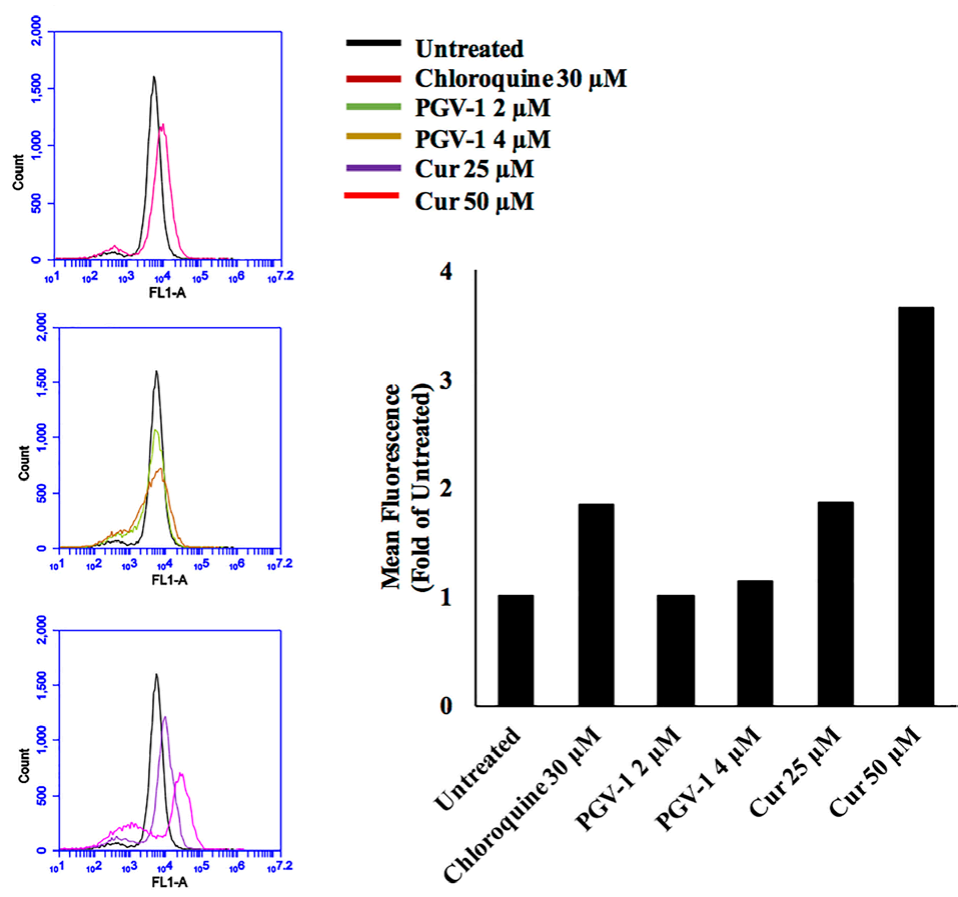
**PGV-1 did not induced Autophagy on 4T1 Cells.**4T1 cells (5x10^4^cells/well) were treated by PGV-1 6 µM for 24h then were detected the presence of autophagy cells by Autophagy detection kit (Abcam). The block graph represents the relative evidence of autophagy positive cells.

Further, we determined the effect of PGV-1 on cell autophagy, a physiological mechanism by which the cell being active to digest the broken materials through generating autophagosomes.^27^ Cells undergoing autophagy resemble the cells in G2/M phase of cell cycle, in which the cells appear as more granulated than the normal cells.^28^ However, we found that PGV-1, in contrast with curcumin, at the cytotoxic concentration did not induce autophagy (Figure 7). Therefore, the anti-proliferative activity of PGV-1 in 4T1 cells is likely through the induction of G2/M arrest and senescence.

### PGV-1 increased the intracellular ROS level

**Figure 8:**
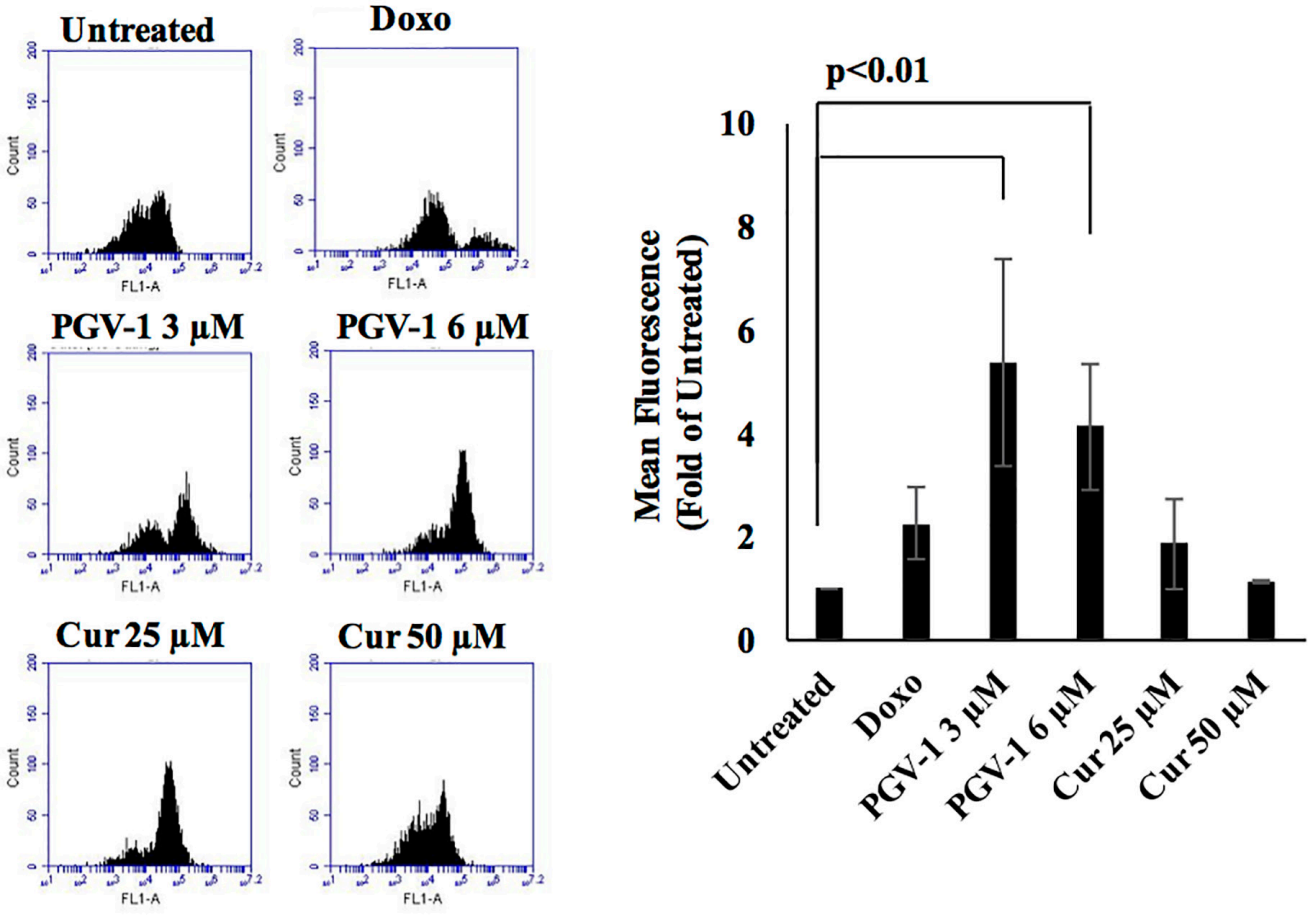
**PGV-1 increases intracellular ROS level in 4T1 cells.** 4T1 cells (5x10^4^cells/well) were treated with either PGV-1 or curcumin for 4h then were subjected to ROS detection analysis by FACS. *P<0.01.

One of important factors regulating senescence is the intracellular ROS level. An increase of intracellular ROS induces cell senescence and activates several anti-apoptotic proteins.^29^ The ROS level is also essential for the stability of growth arrest during the establishment of the senescence cells. During establishment, the genome of cancer cells was in the form of polyploidy resulting on the final form of senescence. However, the higher level of intracellular ROS over the threshold ROS will induce cell death. Therefore, we performed oxidized DCFDA staining flow cytometry to measure intracellular ROS level on 4T1 cells. Since curcumin and PGV-1 themselves give weak fluorescences, we normalized the measurement with an unstained control of each treatment. Our result showed that the treatment of PGV-1 increased intracellular ROS level in 4T1 cells (Figure 8). Moreover, PGV-1 was a stronger ROS inducer compared to curcumin.

### PGV-1 interacts with some ROS metabolizing enzymes

**Figure 9:**
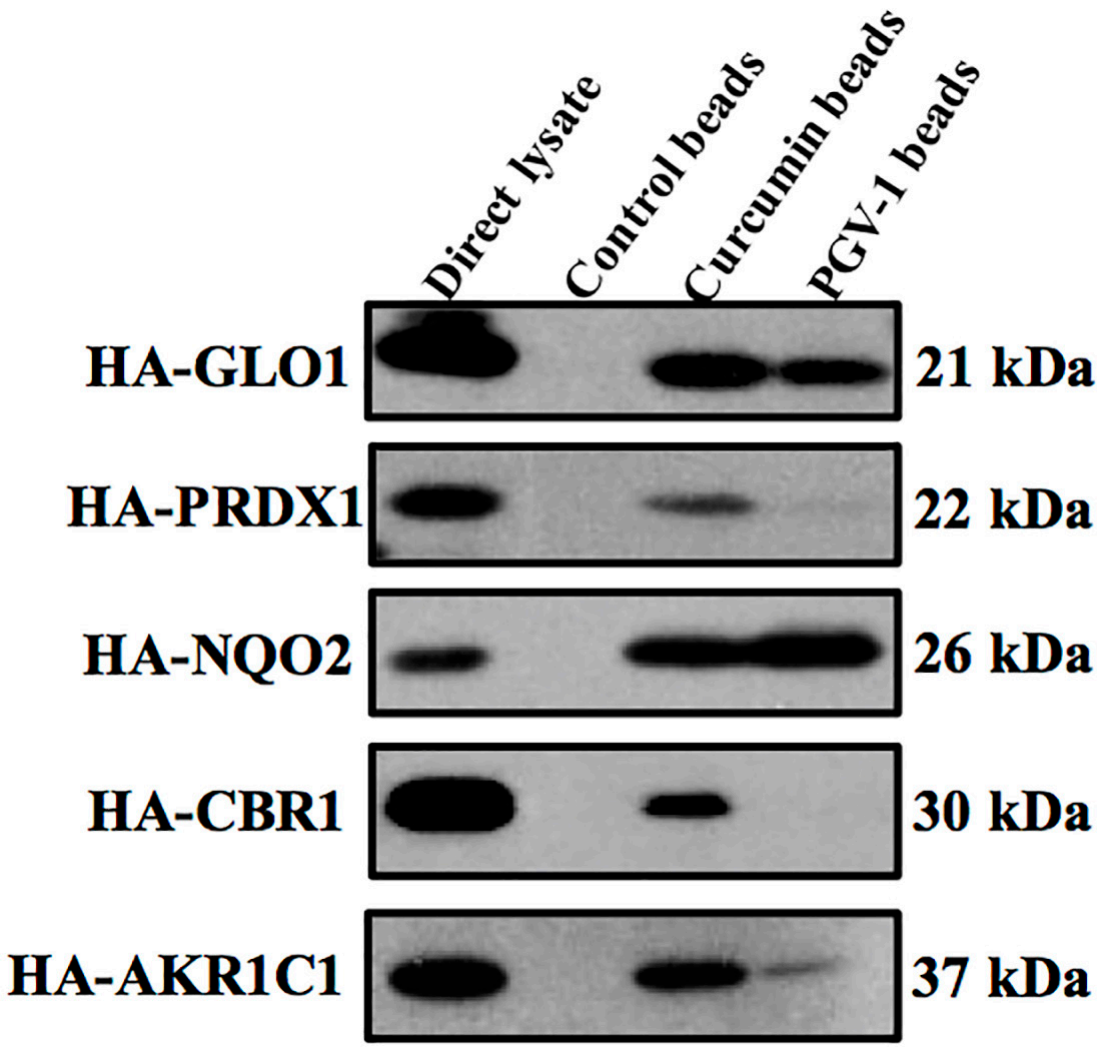
** PGV-1 interacts with several proteins involved in metabolic processes (GLO1, PRDX1, NQO2, AKR1C1).** Curcumin and PGV-1 beads were generated by immobilization on epoxy-sepharose beads as described in the methods. HEK293T cells expressing various HA-tagged proteins (HA-GLO1, HA-PRDX1, HA-NQO2, HA-CBR1, HA-AKR1C1) were incubated with the beads and then subjected to pulldown assay followed by western blot analysis using anti-HA antibody.

A relatively high level of ROS in cancer cells is the consequence of the active metabolism to generate much more energy for the physiological processes. However, ROS level in the cells must be maintained in the physiologically safe level by expressing more highly of ROS metabolizing enzymes. In this regard, we examined the possible interaction between PGV-1 and curcumin with several ROS metabolizing enzymes by pulldown assay continued by western blot on HA-conjugated proteins. We used curcumin as the control of the positive binding.^10^ Interestingly, PGV-1 exhibited shared interaction with curcumin to several enzymes: GLO1, PRDX1, NQO2, and AKR1C1; but not CBR1 (Figure 9). This finding brings us to the conclusion that there is a correlation between the cytotoxic effect of PGV-1 and its inhibitory effect on those ROS metabolizing enzymes.

PGV-1 seems to have less binding affinity to some ROS metabolizing enzymes compared to curcumin. From 5 metabolizing enzymes that finely bind to curcumin, only GLO1 and NQO2 bound tightly to PGV-1. Furthermore, PGV-1 did not bind to CBR1. These results suggested that PGV-1 might possess some other targets that affect the cell senescence and G2/M arrest. The disrupting phenomenon of G2/M leading to the polyploidy may be one of the interesting point to be explored further.

## Conclusion

In conclusion, PGV-1 performs a strong anti-proliferative activity through the induction of G2/M arrest and senescence. The increase of intracellular ROS level by which PGV-1 interact with some ROS metabolizing enzymes plays an important role of its anti-cancer effect. However, other targets related to G2/M arrest and polyploid cells need to be investigated further. Overall, PGV-1 is a promising agent to be developed as an anti-cancer agent for metastatic and malignant cancers.

## Ethical Issues

Not applicable

## Conflict of Interest

We declare no conflict of interest.

## Acknowledgments

We express our gratitude to Indonesian Competence grant and World class Professor program, The Ministry of Research, Technology and Higher Education, Republic of Indonesia who funded this research.

## References

[1] Hanahan D, Weinberg RA (2011). Hallmarks of Cancer: The Next Generation. Cell.

[2] Da’i M, Jenie UA, Supardjan A, Kawaichi M, Meiyanto E (2007). T47D cells arrested at G2M and hyperploidy formation induced by a curcumin’s analogue PGV-1. Indones J Biotechnol.

[3] Da’i M, Suhendi A, Meiyanto E, Jenie UA, Kawaichi M (2017). Apoptosis Induction Effect of Curcumin and Its Analogs Pentagamavunon-0 and Pentagamavunon-1 on Cancer Cell Lines. Asian J Pharm Clin Res.

[4] Meiyanto E, Putri DDP, Susidarti RA, Murwanti R, Sardjiman Sardjiman, Fitriasari A (2014). Curcumin and its analogues (PGV-0 and PGV-1) enhance sensitivity of resistant MCF-7 cells to doxorubicin through inhibition of HER2 and NF-kB activation. Asian Pac J Cancer Prev.

[5] Meiyanto E, Septisetyani EP, Larasati YA, Kawaichi M (2018). Curcumin analog pentagamavunon-1 (PGV-1) sensitizes WiDr Cells to 5-Fluorouracil through Inhibition of NF-κB Activation. Asian Pac J Cancer Prev.

[6] Pulaski BA, Ostrand‐Rosenberg S (2000). Mouse 4T1 breast tumor model. Curr Protoc Immunol.

[7] Bailey-Downs LC, Thorpe JE, Disch BC, Bastian A, Hauser PJ, Farasyn T (2014). Development and characterization of a preclinical model of breast cancer lung micrometastatic to macrometastatic progression. PloS One.

[8] Kumari S, Badana AK, Malla R (2018). Reactive oxygen species: A key constituent in cancer survival Biomark. Insights.

[9] Panieri E, Santoro M (2016). ROS homeostasis and metabolism: a dangerous liason in cancer cells. Cell Death Dis.

[10] Larasati YA, Yoneda-Kato N, Nakamae I, Yokoyama T, Meiyanto E, Kato J (2018). Curcumin targets multiple enzymes involved in the ROS metabolic pathway to suppress tumor cell growth. Sci Rep.

[11] Hong RL, Spohn WH, Hung MC (1999). Curcumin inhibits tyrosine kinase activity of p185neu and also depletes p185neu. Clin Cancer Res.

[12] Shakibaei M, Mobasheri A, Lueders C, Busch F, Shayan P, Goel A (2013). Curcumin enhances the effect of chemotherapy against colorectal cancer cells by inhibition of NF-κB and Src protein kinase signaling pathways. PloS One.

[13] Sardjiman SS, Reksohadiprodjo MS, Hakim L, van der Goot H, Timmerman H (1997). 1,5-Diphenyl-1,4-pentadiene-3-ones and cyclic analogues as antioxidative agents Synthesis and structure-activity relationship. Eur J Med Chem.

[14] Meiyanto E, Supardjan DM, Agustina D (2006). Antiproliferative effect of pentagamavunon-0 on T47D breast cancer cells. Med J Yarsi.

[15] Vyas A, Dandawate P, Padhye S, Ahmad A, Sarkar F (2013). Perspectives on new synthetic curcumin analogs and their potential anticancer properties. Curr Pharm Des.

[16] Nagy LI, Fehér LZ, Szebeni GJ, Gyuris M, Sipos P, Alföldi R (2015). Curcumin and its analogue induce apoptosis in leukemia cells and have additive effects with bortezomib in cellular and xenograft models. BioMed Res Int.

[17] Mock CD, Jordan BC, Selvam C (2015). Recent advances of curcumin and its analogues in breast cancer prevention and treatment. RSC Adv.

[18] Allegra A, Innao V, Russo S, Gerace D, Alonci A, Musolino C (2017). Anticancer activity of curcumin and its analogues: Preclinical and clinical studies. Cancer Invest.

[19] Dhar G, Chakravarty D, Hazra J, Dhar J, Poddar A, Pal M (2015). Actin–curcumin interaction: Insights into the mechanism of actin polymerization inhibition. Biochemistry.

[20] Li W, Jiang Z, Xiao X, Wang Z, Wu Z, Ma Q (2018). Curcumin inhibits superoxide dismutase-induced epithelial-to-mesenchymal transition via the PI3K/Akt/NF-κB pathway in pancreatic cancer cells. Int J Oncol.

[21] Widaryanti B, Meiyanto E, Da’i M, Kawaichi M (2008). PGV-1 is a potent antimitotic agent. Indones J Pharm.

[22] Lee SJ, Langhans SA (2012). Anaphase-promoting complex/cyclosome protein Cdc27 is a target for curcumin-induced cell cycle arrest and apoptosis. BMC Cancer.

[23] Cox CJ, Dutta K, Petri ET, Hwang WC, Lin Y, Pascal SM (2002). The regions of securin and cyclin B proteins recognized by the ubiquitination machinery are natively unfolded. FEBS Lett.

[24] Hermawan A, Fitriasari A, Junedi S, Ikawati M (2011). PGV-0 and PGV-1 increased apoptosis induction of doxorubicin on MCF-7 breast cancer cells. Pharmacon.

[25] Storchova Z, Pellman D (2004). From polyploidy to aneuploidy, genome instability and cancer. Nat Rev Mol Cell Biol.

[26] Campisi J, di Fagagna F d’Adda (2007). Cellular senescence: when bad things happen to good cells. Nat Rev Mol Cell Biol.

[27] Doherty J, Baehrecke E (2018). Life, death and autophagy. Nat Cell Biol.

[28] Pathania AS, Guru SK, Kumar S, Kumar A, Ahmad M, Bhushan S (2016). Interplay between cell cycle and autophagy induced by boswellic acid analog. Sci Rep.

[29] Trachootham D, Alexandre J, Huang P (2009). Targeting cancer cells by ROS-mediated mechanisms: a radical therapeutic approach?. Nat Rev Drug Discov.

